# Cardiovascular Drug Use After Acute Kidney Injury Among Hospitalized Patients With a History of Myocardial Infarction

**DOI:** 10.1016/j.ekir.2022.10.027

**Published:** 2022-11-02

**Authors:** Alejandro Y. Meraz-Muñoz, Nivethika Jeyakumar, Bin Luo, William Beaubien-Souligny, Rahul Chanchlani, Edward G. Clark, Ziv Harel, Abhijat Kitchlu, Javier A. Neyra, Michael Zappitelli, Glenn M. Chertow, Amit X. Garg, Ron Wald, Samuel A. Silver

**Affiliations:** 1 Division of Nephrology, St. Michael’s Hospital, University of Toronto, Ontario, Canada; 2 ICES, Toronto, Ontario, Canada; 3 Division of Nephrology, Center Hospitalier de l’Université de Montréal, Montreal, Quebec, Canada; 4 Division of Pediatric Nephrology, McMaster Children Hospital, McMaster University, Hamilton, Ontario, Canada; 5 Department of Health Research Methods, Evidence, and Impact, McMaster University, Hamilton, Ontario, Canada; 6 Division of Nephrology, Department of Medicine, University of Ottawa, Ottawa, Ontario, Canada; 7 Division of Nephrology, University Health Network, University of Toronto, Toronto, Ontario, Canada; 8 Division of Nephrology, Bone and Mineral Metabolism, University of Kentucky, Lexington, Kentucky, USA; 9 Division of Pediatric Nephrology, Hospital for Sick Children, University of Toronto, Toronto, Ontario, Canada; 10 Division of Nephrology, Stanford University, Palo Alto, California, USA; 11 Division of Nephrology, London Health Sciences Center, Western University, London, Ontario, Canada; 12 Division of Nephrology, Kingston Health Sciences Center, Queen’s University, Kingston, Ontario, Canada

**Keywords:** ACEi, AKI, ARB, ischemic cardiomyopathy, MI

## Abstract

**Introduction:**

Patients who survive acute kidney injury (AKI) may receive fewer cardioprotective drugs. Our objective was to measure the difference in time to dispensing of evidence-based cardiovascular drugs in patients with a history of myocardial infarction (MI) with and without AKI.

**Methods:**

This was a population-based cohort study of patients 66 years of age and older with a history of MI who survived a hospitalization complicated with AKI, propensity-score matched to patients without AKI. The primary outcome was time to outpatient dispensing of an angiotensin-converting enzyme inhibitor (ACEi)/angiotensin II receptor blocker (ARB), statin, or β-blocker within 1 year of hospital discharge.

**Results:**

We identified 28,871 patients with AKI, of whom 21,452 were matched 1:1 to patients without AKI. In the matched cohort, mean age was 80 years, 40% were female, and 34% had an MI during the index hospitalization. AKI was associated with less frequent dispensing of all 3 cardiovascular drug classes within 1 year of hospital discharge (subdistribution hazard ratio [sHR], 0.93; 95% confidence interval [CI], 0.91–0.95). This association was most pronounced in patients with stage 2 (sHR, 0.81; 95% CI, 0.75–0.88) and stage 3 (sHR, 0.71; 95% CI, 0.64–0.79) AKI. We observed less frequent dispensing of statins in patients with stage 2 (sHR, 0.87; 95% CI, 0.81–0.92) and stage 3 (sHR, 0.85; 95% CI, 0.78–0.93) AKI and less frequent dispensing of β-blockers in patients with stage 3 AKI (sHR, 0.86; 95% CI, 0.79–0.94).

**Conclusion:**

In patients with a history of MI, survivors of AKI were less likely to receive prescriptions for ACEi/ARB, statins, or β-blockers within 1 year of hospital discharge. This association was most pronounced in patients with stages 2 and 3 AKI.

## Graphical abstract

**Figure:**
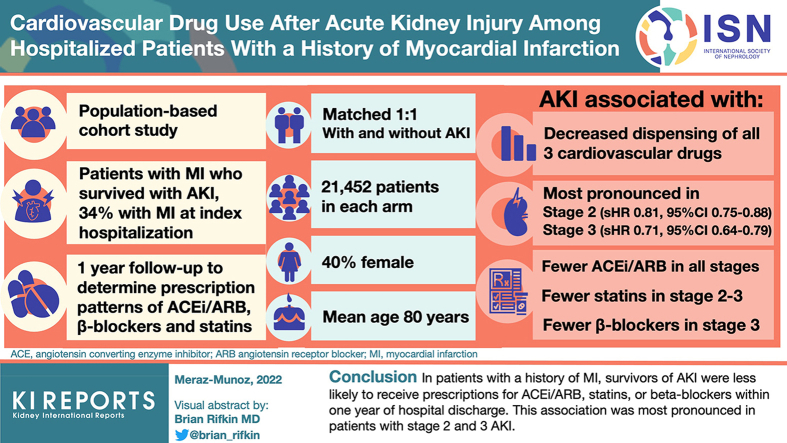

Patients with a history of MI who survive an episode of AKI have a 2-fold risk of death^1^; nevertheless, the reasons for this association are not well understood. Clinical practice guidelines for treating people who experience an MI recommend the long-term use of ACEis or ARBs, β-blockers, and statins^2^^,^^3^; however, patients who develop AKI may experience a disruption in their receipt of evidence-based cardiovascular drugs.^4^^,^^5^ For example, in 158 patients diagnosed with Kidney Disease: Improving Global Outcomes (KDIGO) stage 2 to 3 AKI after a coronary angiography, by 120 days of hospital discharge, only 64%, 73%, and 65% received an ACEi/ARB, β-blocker, and statin, respectively. Conversely, each drug class was prescribed to 83% of patients without AKI.^4^ Delays or interruptions in the use of these drugs in persons with a history of MI have been associated with an increased risk of hospitalizations and death.^6^, ^7^, ^8^

We hypothesized that surviving an episode of AKI is associated with a less frequent outpatient prescription for ACEi/ARB, β-blockers, and statins. Further quantifying this care gap may help inform quality improvement initiatives to ensure safe, timely, and persistent therapy in persons with evidence-based indications after AKI. Therefore, the main objective of this population-based study from Ontario, Canada, was to measure and compare the differences in time with dispensing evidence-based cardiovascular drugs (ACEi/ARB, β-blockers, and statins) in patients with a history of MI with and without AKI. The secondary objectives were to describe this association by AKI severity and the relationship between AKI and dispensing of other cardiovascular medications.

## Methods

### Design

We conducted a population-based cohort study using linked provincial administrative databases in Ontario, Canada (population of 14 million residents). These databases are held at ICES, an independent, nonprofit research institute whose legal status under Ontario’s health information privacy law allows it to collect and analyze health care and demographic data without consent for health system evaluation and improvement. The use of data was authorized under section 45 of Ontario’s Personal Health Information Protection Act, which does not require review by a Research Ethics Board. The reporting of this study follows the Reporting of Studies Conducted Using Observational Routinely Collected Health Data guidelines for observational studies (Supplementary Table S1).^9^ Ontario’s single-payer, universal health care system that encompasses physician services, ambulatory care, and in-hospital care enabled the complete capture of the population, exposure, and outcomes. This study focused on residents ≥66 years old who receive prescription drug coverage through the Ontario Drug Benefit program. Emigration from the province (<0.5% per year) was the only reason for loss of follow-up.^10^

### Databases

We used provincial administrative databases housed at ICES, linked using unique encoded identifiers, to define study criteria, exposures, covariates, and outcomes (Supplementary Tables S2–S4). We ascertained demographics using the Registered Persons Database. We obtained information on hospitalizations using the Canadian Institute for Health Information Discharge Abstract Database, which contains the date of admission and discharge, hospital procedures, and up to 25 diagnoses in the International Classification of Diseases, 10th Revision. The Ontario Health Insurance Plan database contains records of all physician claims for outpatient and inpatient services, including the dates, procedures performed, and diagnoses. Finally, we obtained laboratory data from the Ontario Laboratories Information System, an electronic repository of the province’s laboratory test results. The Ontario Drug Benefit contains all information on outpatient prescription drug dispensing for those 65 years of age and older and is coded with accuracy above 99%.^11^

### Study Population

We assembled a cohort of patients ≥66 years old who survived a hospitalization from January 1, 2008, to March 31, 2017 (the most recent year at the time of analysis to ensure sufficient follow-up time) and a documented history of MI. Notably, the qualifying MI could have occurred at any time before or during the index hospitalization. We ascertained the history of MI with validated International Classification of Diseases codes using an algorithm with a sensitivity of 89% and specificity of 93%.^12^

We excluded patients with a history of maintenance dialysis or kidney transplantation and those without biochemical evidence of AKI who had an administrative code for acute dialysis (likely, dialysis during cardiopulmonary bypass). In addition, to accurately ascertain AKI during the index hospitalization, we excluded hospitalizations in which serum creatinine measurement was not performed and baseline creatinine (defined as the most recent outpatient serum creatinine value 7–365 days before hospital admission) could not be established.^13^^,^^14^

### Exposure

The exposed group comprised patients who developed AKI during the index hospitalization. We defined AKI and its severity using KIDIGO’s serum creatinine criteria.^15^ When a patient had >1 eligible hospitalization, we selected the first eligible hospitalization with AKI and removed all other admissions (i.e., patients could only contribute to a single hospitalization).

### Propensity Score Development

We developed a multivariable logistic regression model to estimate propensity scores for the risk of developing AKI during the index hospitalization.^16^ Clinical significance guided the *a priori* choice of covariates in the model. We included age, sex, rural residence (population ≤10,000 inhabitants), mean income quintile, comorbidities in the preceding 5 years, receipt of different medications in the preceding 4 months, health care utilization in the prior 12 months (i.e., the number of emergency department visits, hospitalizations, and outpatient physician visits), baseline serum creatinine and proteinuria (defined as not done, normal, moderate, or heavy) using a hierarchical combination of albumin/protein-to-creatinine ratio or urinalysis, year of the index hospitalization, index hospitalization details (e.g., admitted to intensive care unit, diagnosis of sepsis, or percutaneous coronary intervention performed), and length of stay (Supplementary Table S5). We used a structured, iterative approach to refine this model and achieve covariate balance within the matched pairs before analyzing the outcomes.^17^ We measured covariate balance by standardized differences (Std diffs) in which an absolute difference >10% represented meaningful imbalance.^18^ We aimed to match each patient with AKI to a patient without AKI 1:1, using a greedy matching algorithm without replacement using the logit of the propensity score (±0.2 SDs).

For different severities of AKI by KDIGO stage, the control group followed their matched pair. To facilitate subgroup comparisons and test for interactions, we forced an exact match on baseline kidney function (estimated glomerular filtration rate ≥60, 45–59, 30–44, <30 ml/min per 1.73 m^2^), use of the combination of the 3 cardiovascular drugs classes (ACEi/ARB, β-blocker, and statin) before hospital admission, congestive heart failure before the index hospitalization, diabetes mellitus at baseline, and MI during the index hospitalization.

### Outcomes

The primary outcome was time to outpatient dispensing of the combination of ACEi/ARB, β-blocker, and statin (all 3 drug classes) in the year after discharge from the index hospitalization. We selected this drug combination as the primary outcome because it is recommended in clinical practice guidelines for most post-MI patients.^2^^,^^3^ Secondary outcomes included time to outpatient dispensing of specific drug classes (drug names listed in Supplementary Table S4), including ACEi/ARB, β-blockers, statins, warfarin, direct anticoagulants, P2Y12 inhibitors, mineralocorticoid receptor antagonists, loop diuretics, thiazides, dihydropyridine calcium channel blockers, biguanides, and nonsteroidal anti-inflammatory inhibitors. We selected these drugs because they are commonly prescribed to patients with cardiovascular disease, or their dosing may be altered or interrupted in the context of AKI.

### Statistical Analyses

We summarized baseline characteristics using descriptive statistics. We expressed continuous variables as the mean (SD) or median (25th, 75th percentile) and categorical variables as proportions. For time-to-event outcomes, we derived sHR and 95% CIs from Fine-Gray models accounting for the competing risk of death using a robust sandwich variance to account for correlation within the matched pairs.^19^

We also performed 2 sensitivity analyses excluding persons who died in ≤90 days of hospital discharge and dementia because cardiovascular drugs may have been appropriately stopped in those patients with short life expectancies. We considered a 2-sided *P* value < 0.05 as statistically significant. We performed all analyses using SAS 9.4 (SAS Institute, Cary, NC).

## Results

### Patient Characteristics

**Figure 1:**
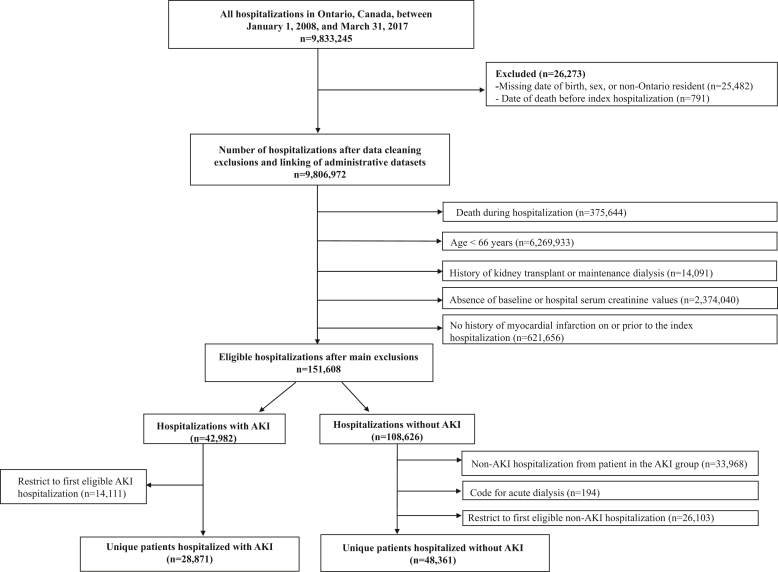
Study flow diagram. AKI, acute kidney injury.

Between January 1, 2008, and March 31, 2017, we identified 28,871 eligible patients who survived a hospitalization complicated with AKI and 48,361 patients without AKI (Figure 1). Of these 77,232 hospitalizations, 26,872 (35%) had an MI during the index hospitalization.

In the unmatched cohort (Supplementary Table S6), patients with AKI were older (80 [8] vs. 78 [8] years, Std diff = 0.19), had more comorbidities, and had more extensive utilization of the health care system in the year before hospital admission. Patients with AKI had lower baseline estimated glomerular filtration rate (52 [22] vs. 65 [20] ml/min per 1.73 m^2^, Std diff = 0.60) and were more likely to have heavy proteinuria (14% vs. 6%, Std diff = 0.30). Patients with AKI were more likely to be using ACEi/ARB (72% vs. 67%, Std diff = 0.11), β-blockers (64% vs. 55%, Std diff = 0.19), or statins (75% vs. 70%, Std diff = 0.10) before their hospital admission. During the index hospitalization, patients with AKI had longer length of stay (14 [25] vs. 8 [15] days, Std diff = 0.31), more sepsis (5% vs. 1%, Std diff = 0.23), and fewer percutaneous coronary interventions (10% vs. 20%, Std diff = 0.27).

We matched 21,452 patients with AKI 1:1 to similar patients without AKI (Table 1). In the AKI group, 17,834 (83%), 2391 (11%), and 1227 (6%) patients experienced KDIGO stages 1, 2, and 3 AKI, respectively, and 256 (1%) received dialysis. The mean discharge serum creatinine was 128 (75) μmol/l (1.4 [0.8] mg/dl), and 7244 patients (34%) had a discharge serum creatinine that exceeded 25% of their prehospital baseline value. The mean follow-up time was 297.3 (122.9) days in the AKI group and 307.9 (114.1) days in the control group. The total person-years of follow-up were 17,642 and 18,083 years in the AKI and control groups, respectively.

**Table 1:** Characteristics of patients aged ≥66 years with a history of myocardial infarction before or during the index hospitalization, propensity-matched on the risk of developing AKI

Baseline characteristics	AKI (*N* = 21,452)	No AKI (*N* = 21,452)	Standardized difference
Demographics, %			
Age (yr), mean (SD)	79.7 (8.0)	80.0 (8.1)	0.03
Female	8701 (40.6)	8631 (40.2)	0.01
Income quintile			
1 (lowest income)	5197 (24.2)	5194 (24.2)	0.00
2	4750 (22.1)	4711 (22.0)	0.00
3 (mid income)	4203 (19.6)	4211 (19.6)	0.00
4	3799 (17.7)	3838 (17.9)	0.00
5 (highest income)	3503 (16.3)	3498 (16.3)	0.00
Rural residence	3208 (15.0)	3240 (15.1)	0.00
Comorbidities in prior 5 yr, %			
Charlson comorbidity index, mean (SD)	1.4 (1.9)	1.5 (1.9)	0.01
Acute dialysis	172 (0.8)	156 (0.7)	0.01
Arrhythmia	5769 (26.9)	5787 (27.0)	0.00
Atrial fibrillation	6087 (28.4)	6087 (28.4)	0.00
Major cancers	4195 (19.6)	4228 (19.7)	0.00
Cerebrovascular disease	3369 (15.7)	3413 (15.9)	0.01
Chronic liver disease	1126 (5.2)	1178 (5.5)	0.01
Chronic obstructive pulmonary disease	2728 (12.7)	2778 (12.9)	0.01
Congestive heart failure	7699 (35.9)	7699 (35.9)	0.00
Coronary artery bypass graft surgery	833 (3.9)	821 (3.8)	0.00
Dementia	5018 (23.4)	5146 (24.0)	0.01
Diabetes	11,398 (53.1)	11,398 (53.1)	0.00
Gastrointestinal bleed	2167 (10.1)	2176 (10.1)	0.00
Hypertension	19,917 (92.8)	20,028 (93.4)	0.02
Pacemaker	2109 (9.8)	2105 (9.8)	0.00
Percutaneous coronary intervention	2998 (14.0)	3001 (14.0)	0.00
Peripheral vascular disease	1091 (5.1)	1099 (5.1)	0.00
Baseline kidney function, %			
Serum creatinine (μmol/l), mean (SD)	110.4 (55.2)	109.1 (54.3)	0.02
Prehospital eGFR (ml/min per 1.73 m^2^)			
≥60	9381 (43.7)	9381 (43.7)	0.00
45–<60	5412 (25.2)	5412 (25.2)	0.00
30–<45	4485 (20.9)	4485 (20.9)	0.00
<30	2174 (10.1)	2174 (10.1)	0.00
Proteinuria			
Normal	6081 (28.3)	5891 (27.5)	0.02
Moderate	2857 (13.3)	2917 (13.6)	0.01
Heavy	2052 (9.6)	2098 (9.8)	0.01
Missing	10,462 (48.8)	10,546 (49.2)	0.01
Medication use in prior 4 mo, %			
Unique drugs prescribed, mean (SD)	11.7 (5.5)	11.8 (5.5)	0.02
ACEi/ARB, statins, and β-blockers	8492 (39.6)	8492 (39.6)	0.00
ACEi/ARB	15,278 (71.2)	15,378 (71.7)	0.01
Statins	15,684 (73.1)	15,644 (72.9)	0.00
β-blockers	13,146 (61.3)	13,206 (61.6)	0.01
Loop diuretics	8229 (38.4)	8382 (39.1)	0.01
Thiazides	4907 (22.9)	4982 (23.2)	0.01
Mineralocorticoid receptor antagonists	1797 (8.4)	1763 (8.2)	0.01
Biguanides	5443 (25.4)	5501 (25.6)	0.01
P2Y12 inhibitors	4928 (23.0)	4957 (23.1)	0.00
Warfarin	3041 (14.2)	3062 (14.3)	0.00
DOACs	2216 (10.3)	2191 (10.2)	0.00
Dihydropyridine calcium blockers	6707 (31.3)	6836 (31.9)	0.01
NSAIDs	2073 (9.7)	2022 (9.4)	0.01
Health care utilization in the prior yr, mean (SD)			
Hospitalizations	1.1 (1.5)	1.1 (1.6)	0.00
Emergency department visits	2.0 (2.7)	2.0 (2.8)	0.00
Nephrology visits	0.3 (0.9)	0.3 (0.9)	0.01
Primary care visits	14.5 (13.7)	14.5 (13.7)	0.00
Cardiology visits	3.7 (5.8)	3.6 (5.6)	0.01
Index hospitalization characteristics, %			
Teaching hospital	7221 (33.7)	7201 (33.6)	0.00
ICU/mechanical ventilation	6640 (31.0)	6743 (31.4)	0.01
CCU admit	1300 (6.1)	1399 (6.5)	0.02
Cardiology involvement	14,974 (69.8)	14,974 (69.8)	0.00
Myocardial infarction	7192 (33.5)	7192 (33.5)	0.00
Percutaneous coronary intervention	2470 (11.5)	2283 (10.6)	0.03
Coronary artery bypass graft surgery	1401 (6.5)	1489 (6.9)	0.02
Pacemaker	1233 (5.7)	1301 (6.1)	0.01
Abdominal aortic aneurysm/aortic bypass repair	85 (0.4)	80 (0.4)	0.00
Major hemorrhage	1028 (4.8)	1040 (4.8)	0.00
Stroke/TIA	1356 (6.3)	1359 (6.3)	0.00
Noncardiac surgery	9902 (46.2)	9746 (45.4)	0.01
Sepsis	644 (3.0)	584 (2.7)	0.02
Length of stay (d), mean (SD)	11.3 (12.8)	10.1 (14.4)	0.09
Discharge potassium (mmol/l), mean (SD)	4.1 (0.5)	4.1 (0.5)	0.09
AKI/kidney details (not included in propensity score)			
AKI severity			
Stage 1	17,834 (83.1)	0 (0.0)	3.14
Stage 2	2391 (11.1)	0 (0.0)	0.50
Stage 3	1227 (5.7)	0 (0.0)	0.35
Discharge serum creatinine (μmol/l), mean (SD)	128.5 (75.2)	99.0 (47.3)	0.47
Discharge eGFR (ml/min per 1.73 m^2^)			
≥60	6694 (31.2)	11,556 (53.9)	0.47
45–<60	5076 (23.7)	4971 (23.2)	0.01
30–<45	5791 (27.0)	3510 (16.4)	0.26
<30	3891 (18.1)	1415 (6.6)	0.36
Dialysis dependence at discharge	108 (0.5)	0 (0.0)	0.10

ACEi, angiotensin-converting enzyme inhibitor; AKI, acute kidney injury; ARB, angiotensin II receptor blocker; CCU, cardiac care unit; DOAC, direct oral anticoagulant; eGFR, estimated glomerular filtration rate; ICU, intensive care unit; NSAIDs, nonsteroidal anti-inflammatory drugs; TIA, transient ischemic attack.

### Outcomes

AKI was associated with less frequent dispensing of the 3 cardiovascular drug classes (ACEi/ARB, β-blocker, and statin) within 1 year of hospital discharge (sHR, 0.93; 95% CI, 0.91–0.95). Dispensing of these drugs was inversely related to KDIGO AKI stage: KDIGO stage 1 (sHR, 0.96; 95% CI, 0.94–0.99), stage 2 (sHR, 0.81; 95% CI, 0.75–0.88), and stage 3 (sHR, 0.71; 95% CI, 0.64–0.79).

**Figure 2:**
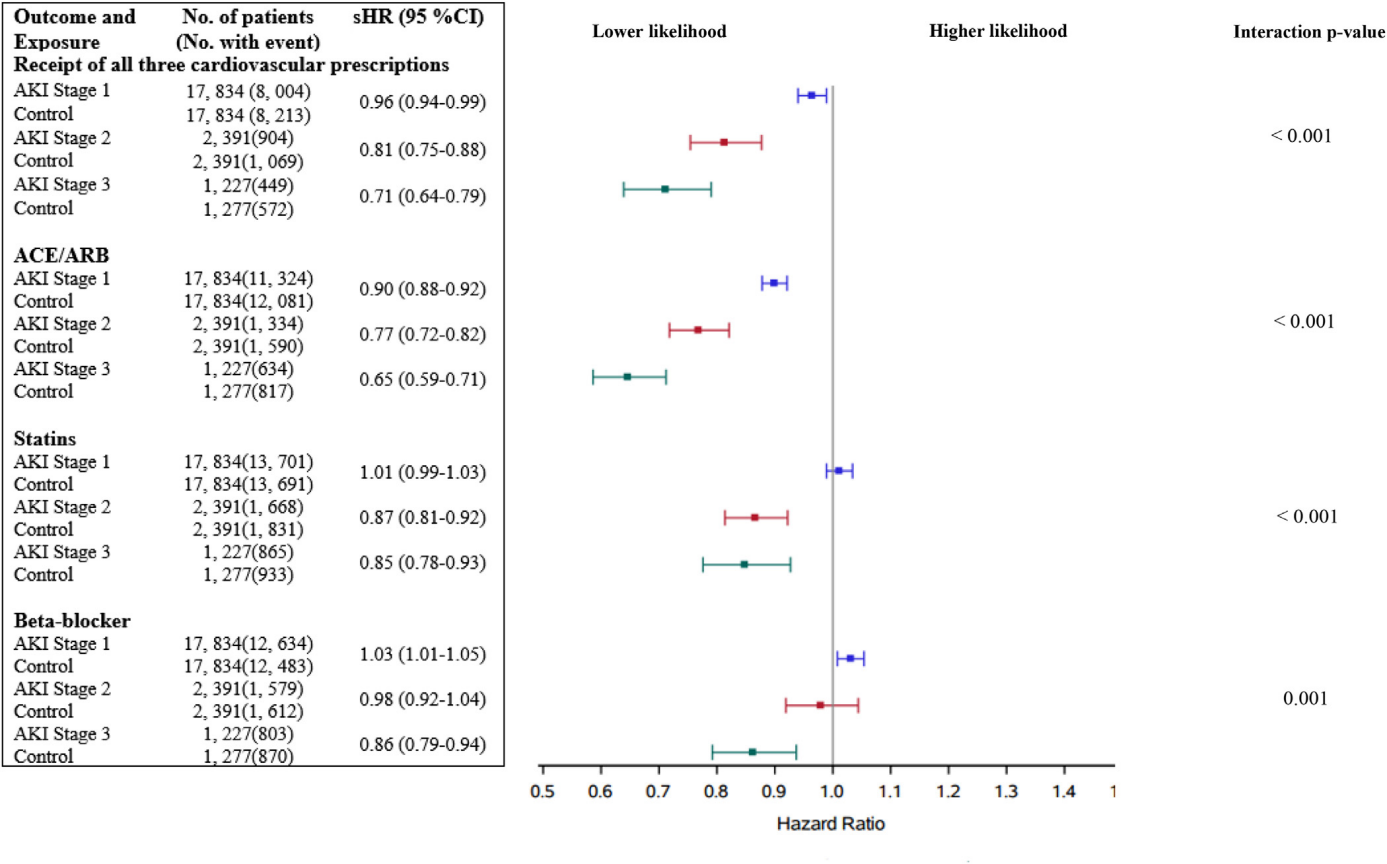
Association of different severities of AKI with the primary outcome and dispensing of ACEi/ARB, β-blocker, and statin. The reference group for each stage of AKI is propensity-matched patients without AKI. ACE, angiotensin-converting enzyme; AKI, acute kidney injury; ARB, angiotensin II receptor blocker; CI, confidence interval; sHR, subdistribution hazard ratio.

For individual drug classes (Figure 2), there was less frequent dispensing of ACEi/ARB in patients with KDIGO stages 1 (sHR, 0.90; 95% CI, 0.88–0.92), 2 (sHR, 0.77; 95% CI, 0.72–0.82), and 3 (sHR, 0.65; 95% CI, 0.59–0.71) AKI. We observed less frequent dispensing of statins in patients with KDIGO stage 2 (sHR, 0.87; 95% CI, 0.81–0.92) and 3 (sHR, 0.85; 95% CI, 0.78–0.93) AKI, and less frequent dispensing of β-blockers in patients with KDIGO stage 3 AKI (sHR, 0.86; 95% CI, 0.79–0.94) within 1 year of hospital discharge.

There were differences in time to dispensing several other medication classes within 1 year of hospital discharge (Table 2 and Supplementary Table S7). AKI was associated with less frequent dispensing of P2Y12 inhibitors (sHR, 0.95; 95% CI, 0.92–0.98) and direct anticoagulants (sHR, 0.94; 95% CI, 0.90–0.98) but more frequent dispensing of warfarin (sHR, 1.16; 95% CI, 1.11–1.22). There was less frequent dispensing of thiazides (sHR, 0.93; 95% CI, 0.89–0.98) and dihydropyridine calcium channel blockers (sHR, 0.89; 95% CI, 0.83–0.95) in patients with AKI and more frequent dispensing of loop diuretics (sHR, 1.20; 95% CI, 1.17–1.23) and mineralocorticoid receptor antagonists (sHR, 1.22; 95% CI, 1.15–1.28). We also observed less frequent dispensing of biguanides (sHR, 0.88; 95% CI, 0.86–0.91) and nonsteroidal anti-inflammatory inhibitors (sHR, 0.89; 95% CI, 0.83–0.95). For most drug classes, the direction of the association was related to the KDIGO AKI stage, that is, the time to dispensing these medications was longer among patients with more severe AKI.

**Table 2:** Association of different severities of AKI with receipt of other drug classes

Outcome and exposure	Events, *n* (%)	Subdistribution hazard ratio (95% CI)	*P* value for interaction
Warfarin			
Stage 1 AKI	3167 (17.8)	1.16 (1.10–1.22)	0.7076
Stage 2 AKI	377 (15.8)	1.17 (1.01–1.35)	
Stage 3 AKI	200 (16.3)	1.26 (1.04–1.54)	
DOAC			
Stage 1 AKI	2891 (16.2)	0.97 (0.92–1.02)	0.0025
Stage 2 AKI	352 (4.7)	0.89 (0.77–1.02)	
Stage 3 AKI	120 (9.8)	0.65 (0.51–0.82)	
P2Y12 inhibitors			
Stage 1 AKI	6483 (36.4)	0.99 (0.96–1.02)	<0.0001
Stage 2 AKI	718 (30.0)	0.81 (0.74–0.89)	
Stage 3 AKI	379 (30.9)	0.71 (0.63–0.80)	
Loop diuretic			
Stage 1 AKI	9639 (54.1)	1.23 (1.20–1.26)	<0.0001
Stage 2 AKI	1145 (47.9)	1.11 (1.03–1.20)	
Stage 3 AKI	521 (42.5)	0.93 (0.83–1.04)	
Thiazide diuretic			
Stage 1 AKI	2896 (16.2)	0.97 (0.92–1.02)	0.0023
Stage 2 AKI	324 (13.6)	0.82 (0.71–0.94)	
Stage 3 AKI	138 (11.3)	0.70 (0.56–0.86)	
Mineralocorticoid receptor antagonist			
Stage 1 AKI	2461 (13.8)	1.24 (1.17–1.32)	0.0553
Stage 2 AKI	296 (12.4)	1.16 (0.98–1.36)	
Stage 3 AKI	117 (9.5)	0.92 (0.73–1.18)	
DHP calcium channel blocker			
Stage 1 AKI	5056 (28.4)	0.95 (0.91–0.98)	0.0429
Stage 2 AKI	594 (24.8)	0.82 (0.74–0.91)	
Stage 3 AKI	360 (29.3)	0.91 (0.80–1.05)	
Biguanide			
Stage 1 AKI	3939 (22.1)	0.91 (0.88–0.94)	0.0002
Stage 2 AKI	495 (20.7)	0.84 (0.76–0.92)	
Stage 3 AKI	232 (18.9)	0.68 (0.59–0.78)	
NSAID			
Stage 1 AKI	1263 (7.1)	0.90 (0.84–0.97)	0.6015
Stage 2 AKI	157 (6.6)	0.86 (0.70–1.06)	
Stage 3 AKI	65 (5.3)	0.77 (0.56–1.06)	

AKI, acute kidney injury; CI, confidence interval; DHP, dihydropyridine; DOAC, direct oral anticoagulant; NSAID, nonsteroidal anti-inflammatory drug.

The reference group for each stage of AKI is propensity-matched patients without AKI.

**Figure 3:**
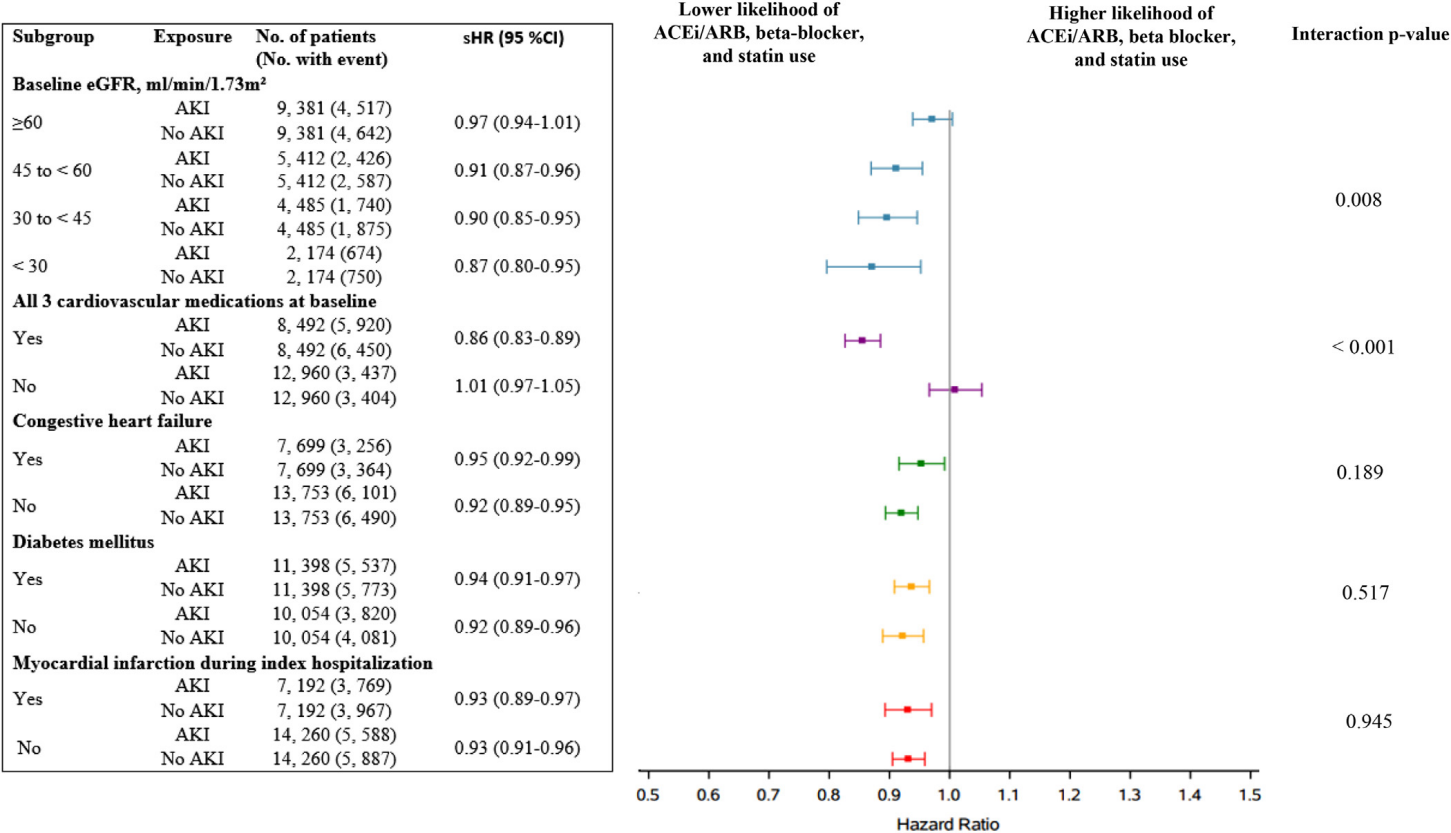
Association of AKI with dispensing of an ACEi/ARB, β-blocker, and statin (all 3 drugs) within 1 year of hospital discharge, stratified by prespecified subgroups. ACEi, angiotensin-converting enzyme inhibitor; AKI, acute kidney injury; ARB, angiotensin II receptor blocker; CI, confidence interval; eGFR, estimated glomerular filtration rate; sHR, subdistribution hazard ratio.

In the prespecified subgroups (Figure 3), the time to dispensing of all 3 drugs (ACEi/ARB, β-blocker, and statin) differed based on baseline estimated glomerular filtration rate (*P* = 0.008 for interaction) and patients taking all 3 drugs at baseline (*P* < 0.001 for interaction). The association of ACEi/ARB, β-blocker, and statin dispensing after AKI did not differ based on diabetes mellitus, preexisting heart failure, or the occurrence of MI during the index hospitalization. In the 2 sensitivity analyses, the results resembled the primary analysis when we excluded patients who died ≤90 days of hospital discharge (sHR, 0.96; 95% CI, 0.94–0.98) and patients with dementia (sHR, 0.94; 95% CI, 0.91–0.96). In a *post hoc* analysis, individual drug prescription of ACEi/ARB, β-blockers, or statins was more persistent with less discontinuation in patients without history of AKI (Supplementary Table S8).

## Discussion

In patients aged ≥66 years with a history of MI, AKI was associated with less frequent dispensing of the combination of ACEi/ARB, β-blocker, and statin within 1 year of hospital discharge. The foremost driver of this finding was the less frequent dispensing of ACEi/ARBs, even in patients with KDIGO stage 1 AKI. However, statins and β-blockers were less likely to be dispensed to patients with stage 2 to 3 AKI and stage 3 AKI, respectively. These results highlight a pivotal opportunity to improve care after hospitalization with AKI.

This care gap is consistent with previous work in patients who underwent coronary angiography that demonstrated a lower likelihood of ACEi/ARB use across all stages of AKI, that was more pronounced in 158 patients with KDIGO stage 2 to 3 AKI, along with less use of β-blockers and statins only in patients with severe AKI.^4^ Our findings in >3500 patients with KDIGO stage 2 to 3 AKI showed that only 55%, 65%, and 70% of patients received an ACEi/ARB, β-blocker, or statin, respectively, at 1 year of hospital discharge, corresponding with the prescription rates of 64%, 73%, and 65% observed at after coronary angiography,^4^ and extend these results to a contemporary population more removed from their MI.

We suspect the association with ACEi/ARB use may be explained by assuming that these drugs impair glomerular autoregulation and contribute to recurrent or persistent AKI.^20^ However, 3 cohort studies found that ACEi/ARB use was not associated with recurrent AKI.^5^^,^^21^^,^^22^ These findings suggest that preventing recurrent AKI should not be the sole reason to avoid ACEi/ARB in patients with indications for these drugs. Failure to continue or to initiate ACEi/ARB for secondary prevention in patients with a history of MI out of fear of worsening kidney function may contribute to adverse outcomes in these high-risk patients.^23^ Regarding β-blockers and statins, we suspect underutilization in severe AKI could be related to concerns over hemodynamic instability or toxicity from low kidney function.^24^^,^^25^ Failure to continue or initiate these drugs provides another example of how kidney function modifies physician behavior and prescription patterns,^26^ even 1 year after an AKI episode.

In addition, our study examined the time to dispensing other cardiovascular medications within 1 year of discharge. We found that AKI was associated with less frequent dispensing of P2Y12 inhibitors, likely due to bleeding concerns and less coronary revascularization in patients with kidney disease.^27^^,^^28^ AKI was associated with increased loop diuretics and mineralocorticoid receptor antagonists dispensing, but this association was no longer present in KDIGO stage 3 AKI. We suspect this relationship may be a marker of worsening heart failure in patients with AKI (i.e., cardiorenal syndrome) that necessitated more diuresis postdischarge because patients with AKI have a decreased ability to maintain an adequate volume status and often need higher doses or a combination of diuretics.^29^ However, increasing diuretics and other antihypertensives may have been avoided for safety concerns in some patients with severe kidney dysfunction (i.e., KDIGO stage 3), potentially because of low blood pressure and concerns that these drugs would not be tolerated. These results support observations from previous literature that patients have an increased risk for hospitalization with heart failure after AKI that increases by AKI severity,^21^^,^^30^^,^^31^ making the less frequent dispensing of loop diuretics, ACEi/ARB, and β-blockers in patients with severe AKI even more concerning.

Another important observation is that patients with baseline use of ACEi/ARB, β-blocker, and statin were less likely to receive these drugs in the subsequent year of follow-up if they had AKI. Possibly these drugs were stopped during the AKI episode and not restarted out of an abundance of precaution, indicative of heterogeneous practice patterns post-AKI.^4^^,^^5^^,^^23^^,^^32^ This association provides another example of “renalism,” whereby beneficial interventions are underutilized in patients with kidney disease out of misplaced or exaggerated harm concerns.^28^ The current practice is unlikely to change without system-based solutions to overcome these barriers and optimize evidence-based medications after AKI, similar to the American Heart Association Get With The Guidelines initiative.^33^

Our findings highlight the degree to which AKI survivors are deprived of critical therapies known to improve outcomes in patients with coronary artery disease. The use of ACEi/ARBs should be strongly considered for most patients with coronary artery disease, especially those with reduced ejection fraction and hypertension.^34^^,^^35^ Although there may be some legitimate safety concerns with ACEi/ARBs in a small number of patients post-AKI, these kidney-related anxieties should not extend to patients with evidence-based indications for β-blockers or statins. This study should also help inform the design of clinical trials that test follow-up care after AKI^36^ and guide knowledge translation material and quality improvement efforts that target primary care providers, cardiologists, nephrologists, and pharmacists on how to manage patients after hospitalization with AKI.^37^^,^^38^

This study has several strengths. First, we identified more than 42,000 matched patients with a previous history of MI across Ontario, Canada, including more than 3500 patients with KDIGO stage 2 to 3 AKI. Second, the linked health care databases had little missing data and virtually complete patient follow-up. Third, this cohort was well characterized regarding sociodemographic characteristics, comorbidities, and kidney function, including comprehensive medication information and serum creatinine results, allowing for adjusting potential confounders. Finally, this study provided a current estimate of drug use after AKI, during an era when more attention has been devoted to formal medication reconciliation programs and care transitions.

This study also has limitations. The results are from a single Canadian province with universal, single-payer health care and limited to patients 66 years or older; therefore, the results may not apply to other jurisdictions or populations. Similar to other observational studies in this area, we could not determine the specific indication for drug initiation or whether drug use was appropriate or inappropriate.^39^ For example, ACEi/ARB may not be indicated in all patients post-MI (e.g., patients without hypertension or heart failure with reduced left ventricular function),^3^ and some medications may have been reasonably stopped for hyperkalemia or avoided in patients with different goals of care. However, discharge potassium was included in the propensity score, and the results remained consistent when patients with short life expectancies were excluded. Conditioning on the availability of baseline serum creatinine may also have selected for a sicker cohort more likely to receive medications; however, even in this group, prescription of all 3 drugs at 1 year was only 43.6%. Finally, there is likely to be residual confounding because administrative data limit our ability to capture all comorbid conditions and their severity, such as the type of heart failure (preserved or reduced ejection fraction), AKI etiology, blood pressure, and volume status. Using propensity score matching should reduce the degree of confounding but cannot eliminate it.

In this population-based study of >77,000 patients with a prior history of MI, we demonstrated that patients who survive a hospitalization complicated with AKI are less likely to receive ACEi/ARB across all stages of AKI within the following year after hospital discharge. In addition, patients with severe AKI were also less likely to receive β-blockers and statins during the same time frame. Considering the importance of appropriate pharmacotherapy in patients with cardiovascular disease and AKI, these results should be confirmed and quantified in other regions, ideally with more granular information on reasons for discontinuation after AKI. These findings should also inform educational strategies and clinical pathways to help improve transitions in care for this vulnerable and growing patient population.

## Disclosure

All authors have completed the ICMJE uniform disclosure form at www.icmje.org/coi_disclosure.pdf. SAS has received speaking fees from Baxter Canada. The remaining authors declare no support from any organization for the submitted work: no financial relationships with any organizations that might have an interest in the submitted work in the previous 3 years and no other relationships or activities that could appear to have influenced the submitted work.

## Supplementary Material

Supplementary File (PDF)

**Table S1.** Checklist of items from the Reporting of Studies Conducted Using Observational Routinely Collected Health Data guidelines for observational studies.

**Table S2.** Administrative data codes used to identify cohort of individuals hospitalized with and without AKI.

**Table S3.** Administrative data codes used to define baseline characteristics.

**Table S4.** Drugs from the Ontario Drug Benefit database used to identify outcomes.

**Table S5.** Variables included in the propensity score model.

**Table S6.** Unmatched characteristics of patients aged ≥66 years old who survived an inpatient hospitalization with a history of myocardial infarction before or during the index hospitalization.

**Table S7.** Association of different severities of AKI with receipt of other drug classes. The reference group for each stage of AKI is propensity-matched patients without AKI.

**Table S8.** Drug persistence and discontinuation at 1 year.
