# Supplementary material for: Cardiovascular Drug Use After Acute Kidney Injury Among Hospitalized Patients With a History of Myocardial Infarction
Source: Kidney Int Rep. 2022 Nov 2;8(2):294–304. doi: 10.1016/j.ekir.2022.10.027 (PMC9939314; doi:10.1016/j.ekir.2022.10.027)
Supplement: Supplementary File (PDF) [file mmc1.pdf]

Supplemental Material—Table of Contents

Supplementary Table S1: Checklist of items from the Reporting of Studies Conducted Using Observational Routinely Collected Health Data guidelines for observational studies.

Supplementary Table S2: Administrative data codes used to identify cohort of individuals hospitalized with and without AKI.

Supplementary Table S3: Administrative data codes used to define baseline characteristics.

Supplementary Table S4: Drugs from the Ontario Drug Benefit (ODB) database used to identify outcomes.

Supplementary Table S5: Variables included in the propensity score model.

Supplementary Table S6: Unmatched characteristics of patients aged ≥ 66 years old who survived an inpatient hospitalization with a history of myocardial infarction prior to or during the index hospitalization.

Supplementary Table S7: Association of different severities of AKI with receipt of other drug classes. The reference group for each stage of AKI is propensity-matched patients without AKI.

Supplementary Table S8: Drug persistence and discontinuation at one-year.

Supplementary Table S1: Checklist of items from the Reporting of Studies Conducted Using Observational Routinely Collected Health Data guidelines for observational studies

	Item No	STROBE items	RECORD items	Reported
**Title and abstract**	1	(a) Indicate the study's design with a commonly used term in the title or the abstract.<br>(b) Provide in the abstract an informative and balanced summary of what was done and what was found.	(1.1) The type of data used should be specified in the title or abstract. When possible, the name of the databases used should be included.<br>(1.2) If applicable, the geographic region and time frame within which the study took place should be reported in the title or abstract.<br>(1.3) If linkage between databases was conducted for the study, this should be clearly stated in the title or abstract.	Title and Abstract
**Introduction**				
Background/ rationale	2	Explain the scientific background and rationale for the investigation being reported.		Background
Objectives	3	State specific objectives, including any prespecified hypotheses.		Background
**Methods**				
Study design	4	Present key elements of study design early in the paper.		Methods: Study Design
Setting	5	Describe the setting, locations, and relevant dates, including periods of recruitment, exposure, follow-up, and data collection.		Methods: Study Design, Databases, Study Population, Exposure
Participants	6	(a) Give the eligibility criteria, and the sources and methods of selection of participants. Describe methods of follow-up.<br>(b) For matched studies, give matching criteria and number of exposed and unexposed.	(6.1) The methods of study population selection (such as codes or algorithms used to identify subjects) should be listed in detail. If this is not possible, an explanation should be provided.<br>(6.2) Any validation studies of the codes or algorithms used to select the population should be referenced. If validation was conducted for this study and not published elsewhere, detailed methods and results should be provided.<br>(6.3) If the study involved linkage of databases, consider use of a flow diagram or other graphical display to demonstrate the data linkage process, including the number of individuals with linked data at each stage.	Methods: Study Population, Exposure, and Propensity Score Development<br><br>Supplemental Table 2<br><br>Results: Patient Characteristics<br><br>Figure 1
Variables	7	Clearly define all outcomes, exposures, predictors, potential confounders, and effect modifiers. Give diagnostic criteria, if applicable.	(7.1) A complete list of codes and algorithms used to classify exposures, outcomes, confounders, and effect modifiers should be provided. If these cannot be reported, an explanation should be provided.	Methods: Exposure, Outcome, and Propensity Score Development

				Supplemental: Tables 2, 3, and 4
Data sources/ measurement	8	For each variable of interest, give sources of data and details of methods of assessment (measurement). Describe comparability of assessment methods if there is more than one group.		Supplemental: Tables 2, 3, and 4
Bias	9	Describe any efforts to address potential sources of bias.		Methods: Propensity Score Development
Study size	10	Explain how the study size was arrived at.		Methods: Study Population<br>Figure 1
Quantitative variables	11	Explain how quantitative variables were handled in the analyses. If applicable, describe which groupings were chosen and why.		Statistical Analyses
Statistical methods	12	(a) Describe all statistical methods, including those used to control for confounding.<br>(b) Describe any methods used to examine subgroups and interactions.<br>(c) Explain how missing data were addressed.<br>(d) If applicable, explain how loss to follow-up was addressed.<br>(e) Describe any sensitivity analyses.		Statistical Analyses
Data access and cleaning methods		N/A	(12.1) Authors should describe the extent to which the investigators had access to the database population used to create the study population.<br>(12.2) Authors should provide information on the data cleaning methods used in the study.	Methods: Databases<br>Data access/access to data analysis protocol
Linkage		N/A	(12.3) State whether the study included person-level, institutional-level, or other data linkage across two or more databases. The methods of linkage and methods of linkage quality evaluation should be provided.	Methods: Databases
**Results**				
Participants	13	(a) Report numbers of individuals at each stage of study--e.g. numbers potentially eligible, examined for eligibility, confirmed eligible, included in the study, completing follow-up, and analyzed.<br>(b) Give reasons for non-participation at each stage.<br>(c) Consider use of a flow diagram.	(13.1) Describe in detail the selection of the persons included in the study (i.e., study population selection), including filtering based on data quality, data availability, and linkage. The selection of included persons can be described in the text and/or by means of the study flow diagram.	Results: Patient Characteristics<br>Figure 1

Descriptive data	14	(a) Give characteristics of study participants (e.g. demographic, clinical, social) and information on exposures and potential confounders.<br>(b) Indicate number of participants with missing data for each variable of interest.<br>(c) Summarize follow-up time (e.g. average and total amount).		Results: Patient Characteristics<br>Table 1<br>Supplemental Table 6
Outcome data	15	Report numbers of outcome events or summary measures over time.		Results: Outcomes<br>Table 2
Main results	16	(a) Give unadjusted estimates and, if applicable, confounder-adjusted estimates and their precision (e.g. 95% confidence interval). Make clear which confounders were adjusted for and why they were included.<br>(b) Report category boundaries when continuous variables were categorized.<br>(c) If relevant, consider translating estimates of relative risk into absolute risk for a meaningful time period.		Results: Outcomes<br>Table 2
Other analyses	17	Report other analyses done (e.g. analyses of subgroups and interactions, and sensitivity analyses).		Results: Outcomes<br>Figure 2
Key results	18	Summarize key results with reference to study objectives.		Discussion
Limitations	19	Discuss limitations of the study, taking into account sources of potential bias or imprecision. Discuss both direction and magnitude of any potential bias.	(19.1) Discuss the implications of using data that were not created or collected to answer the specific research question(s). Include discussion of misclassification bias, unmeasured confounding, missing data, and changing eligibility over time, as they pertain to the study being reported.	Discussion
Interpretation	20	Give a cautious overall interpretation of results considering objectives, limitations, multiplicity of analyses, results from similar studies, and other relevant evidence.		Discussion
Generalizability	21	Discuss the generalizability (external validity) of the study results.		Discussion
**Other information**				
Funding	22	Give the source of funding and the role of the funders for the present study and, if applicable, for the original study on which the present article is based.		Funding

Accessibility of protocol, raw data, and programming code	N/A	(22.1) Authors should provide information on how to access any supplemental information such as the study protocol, raw data, or programming code.	Data access/access to data analysis protocol

Supplementary Table S2: Administrative data codes used to identify cohort of individuals hospitalized with and without AKI

Database	Variable	Code or Algorithm
**Inclusion Criteria**		
Hospital Admission		
CIHI-DAD	ADMDATE	N/A
	EPI	
	EPIFLAG	
	EPIVISIT	
**Exclusion Criteria**		
Invalid or missing sex, age, or unique identifying number, non-Ontario resident		
RPDB		% getdemo ICES macro
Death on or before index date		
RPDB	DTHDATE	N/A
Age < 66 years		
RPDB	AGE	N/A
Absence of outpatient referent serum creatinine values and inpatient values during episode of care		
OLIS	OBSERVATIONCODE	"14682-9"
Kidney transplant prior to index date		
CORR RECIPIENT_TREATMENT	TREATMENT_CODE	"181"
	TRANSPLANTED_ORGAN _TYPE_CODE	"10", "11", "12", "18", "19"
Chronic dialysis prior to index date		
CORR RECIPIENT_TREATMENT	TREATMENT_CODE	not equal to "171", "181"
Restrict to patients with a history of myocardial infarction on or prior to episode of care		
CIHI-DAD	ICD9	"410"
	ICD10	"I21", "I22"
**AKI Specific Exclusion (to identify patients without AKI)**		
Does not meet AKI KDIGO definition		
OLIS	OBSERVATIONCODE	"14682-9"
Acute dialysis during episode of care		
OHIP	FEECODE	"R849", "G323", "G866", "G330", "G331", "G093", "G095", "G294", "G295"

Abbreviations: CIHI-DAD: Canadian Institutes for Health Information's Discharge Abstract Database, CORR: Canadian Organ Replacement Registry, OHIP: Ontario Health Insurance Plan, OLIS: Ontario Laboratory Information System, RPDB: Registered Persons Database

Supplementary Table S3: Administrative data codes used to define baseline characteristics

Database	Variable	Code or Algorithm
Age		
RPDB	AGE	N/A
Sex		
RPDB	SEX	N/A
Income quintile		
RPDB	INCQUINT	N/A
Rural residence		
RPDB	RURAL	N/A
Charlson comorbidity index		
CIHI-DAD/SDS	ICD10	%charlson macro
Acute Dialysis		
OHIP	FEECODE	"R849", "G323", "G866", "G330", "G331", "G093", "G095", "G294", "G295"
Arrhythmia		
CIHI-DAD/SDS	ICD10	"I48", "I44", "I45", "I47", "I4900", "I4901", "I491", "I492", "I493", "I494", "I498", "I499", "R000" "R001"
OHIP	FEECODE	"G178", "G179", "G249", "G261", "G259", "Z443", "Z431", "Z437"
Atrial Fibrillation		
CIHI-DAD/SDS	ICD10	"I48"
OHIP	DXCODE	"427"
Major Cancers		
CIHI-DAD/SDS	ICD10	"971", "980", "982", "984", "985", "986", "987", "988", "989", "990", "991", "993", "C15", "C18", "C19", "C20", "C22", "C25", "C34", "C50", "C56", "C61", "C82", "C83", "C85", "C91", "C92", "C93", "C94", "C95", "D00", "D05", "D010", "D011", "D012", "D022", "D075"
OHIP	DXCODE	"203", "204", "205", "206", "207", "208", "150", "154", "155", "157", "162", "174", "175", "183", "185"
Cerebrovascular Disease		
CIHI-DAD/SDS	ICD10	"I60", "I600", "I601", "I602", "I603", "I604", "I605", "I606", "I607", "I608", "I609", "I61", "I610", "I611", "I612", "I613", "I614", "I615", "I616", "I618", "I619", "I630", "I631", "I632", "I633", "I634", "I635", "I638", "I639", "I64", "H341", "G450", "G451","G452", "G453", "G458", "G459","H340"

OHIP	DXCODE	"436", "432", "435"
Chronic Liver Disease		
CIHI-DAD/SDS	ICD10	"B16", "B17", "B18", "B19", "I85", "R17", "R18", "R160", "R162", "B942", "Z225", "E831", "E830", "K70", "K713", "K714", "K715", "K717", "K721", "K729", "K73", "K74", "K753", "K754", "K758", "K759", "K76", "K77"
OHIP	DXCODE	"571", "573", "070"
	FEECODE	"Z551", "Z554"
Chronic Obstructive Pulmonary Disease		
CIHI-DAD/SDS	ICD10	"J41", "J43", "J44"
Congestive Heart Failure		
CIHI-DAD/SDS	ICD10	"I099", "I420", "I425", "I426", "I427", "I428", "I429", "I43", "I500", "I501", "I509", "I255", "J81"
	CCI	"1HP53", "1HP55", "1HZ53GRFR", "1HZ53LAFR", "1HZ53SYFR"
OHIP	DXCODE	"428"
	FEECODE	"R701", "R702", "Z429"
Coronary Artery Bypass Graft Surgery		
CIHI-DAD/SDS	CCI	"1IJ76"
OHIP	FEECODE	"R742", "R743", "E654", "E645", "E652", "E646"
Dementia		
CIHI-DAD/SDS	ICD10	"F065", "F066", "F068", "F069", "F09", "F00", "F01", "F02", "F03", "F051", "G30", "G31", "R54"
OHIP	DXCODE	"290","331", "797"
Diabetes		
CIHI-DAD/SDS	ICD10	"E10", "E11", "E13", "E14"
OHIP	DXCODE	"250"
	FEECODE	"K045", "K046", "K029", "K030", "Q040"
Gastrointestinal Bleed		
CIHI-DAD/SDS	ICD10	"I850", "I9820", "I983", "K2210", "K2211", "K2212", "K2214", "K2216", "K226", "K228", "K250", "K252", "K254", "K256", "K260", "K262", "K264", "K266", "K270", "K272", "K274", "K276", "K280", "K282", "K284", "K286", "K290", "K3180", "K6380", "K920", "K921", "K5520", "K625", "K922"
Hypertension		
CIHI-DAD/SDS	ICD10	"I10", "I11", "I12", "I13", "I15"
OHIP	DXCODE	"401", "402", 403"
Pacemaker		

CIHI-DAD/SDS	CCI	"1HZ37", "1HD53GRJA", "1HD54GRJA", "1HZ53GRNK", "1HZ53GRNL", "1HZ53GRNM", "1HZ54LANJ", "2HZ07NK", "2HZ07NL", "2HZ07NM", "1HZ53GRFR", "1HZ53LAFR", "1HZ53SYFR", "1HD55", "1HZ09", "1HZ55", "2HZ24", "1HZ53GRNN"
OHIP	FEECODE	"G303", "Z433", "Z435", "Z443", "Z444", "Z445", "R752", "Z412", "Z428", "E628", "G176", "G177", "G115"
Percutaneous Coronary Intervention		
CIHI-DAD/SDS	CCI	"1IJ50", "1IJ57GQ", "1IJ54GQAZ"
OHIP	FEECODE	"Z434", "G262", "G298"
Peripheral Vascular Disease		
CIHI-DAD/SDS	ICD10	"I700", "I702", "I708", "I709", "I731", "I738", "I739", "K551"
	CCI	"1KA76", "1KA50", "1KE76", "1KG50", "1KG57", "1KG76MI", "1KG87", "1IA87LA", "1IB87LA", "1IC87LA", "1ID87", "1KA87LA", "1KE57"
OHIP	FEECODE	"R787", "R780", "R797", "R804", "R809", "R875", "R815", "R936", "R783", "R784","R785", "E626", "R814", "R786", "R937", "R860", "R861", "R855", "R856", "R933", "R934", "R791", "E672", "R794", "R813", "R867", "E649"
Sepsis		
CIHI-DAD/SDS	ICD10	"A021", "A392", "A393", "A394", "A400", "A401", "A402", "A408", "A409", "A410", "A411", "A412", "A403", "A414", "A4159", "A413", "A4150", "A4151", "A4152", "A4158", "A4180", "A4188", "A427", "A419"
Abdominal Aortic Aneurysm/ Aortic Bypass Repair		
CIHI-DAD/SDS	CCI	"1KA76"
OHIP	FEECODE	"R802", "R816", "R817", "R783", "R784", "R785", "R814"
Non-Cardiac Surgery		
OHIP	FEECODE	Available upon request due to large number of codes
Major Hemorrhage		
CIHI-DAD/SDS	ICD10	"I600", "I601", "I602", "I603", "I604", "I605", "I606", "I607", "I609", "I61", "I62", "I850", "I9820", "I983", "K2210", "K2211", "K2212", "K2214", "K2216", "K226", "K228", "K250", "K252", "K254", "K256", "K260", "K262", "K264", "K266", "K270", "K272", "K274", "K276", "K280", "K282", "K284", "K286", "K290", "K3180", "K6380", "K920", "K921", "K5520", "K625", "K922", "M2509", "M2501", "M2502", "M2503", "M2504", "M2505", "M2506", "M2507", "M2508", "M2500", "M1229", "M1221", "M1222", "M1223", "M1224", "M1225", "M1226", "M1227", "M1228", "M1220", "R58"
Stroke/Transient Ischemic Attack		

CIHI-DAD/SDS	ICD10	I60", "I600", "I601", "I602", "I603", "I604", "I605", "I606", "I607", "I608", "I609", "I61", "I610", "I611", "I612", "I613", "I614", "I615", "I616", "I618", "I619", "I630", "I631", "I632", "I633", "I634", "I635", "I638", "I639", "I64", "H341", "G450", "G451","G452","G453","G458","G459","H340"
ICU/Mechanical Ventilation		
CIHI-DAD/SDS	CCI	"1GZ31CAND", "1GZ31CRND", "1GZ31GPND"
OHIP	FEECODE	"G557", "G558", "G559", "G405", "G406", "G407" , "G400", "G401", "G402"
Serum Creatinine		
OLIS	OBSERVATIONCODE	"14682-9"
Proteinuria		
OLIS	OBSERVATIONCODE	"14959-1", "30000-4", "32294-1", "34366-5", "5804-0", "50561-0"
Hospitalizations		
CIHI-DAD/SDS	ADMDATE	N/A
Emergency Department Visits		
NACRS	REGDATE	N/A
Nephrology Visits		
IPDB	MAINSPECIALTY	"NEPHROLOGY"
OHIP	SPEC	"16"
	FEECODE	"A160", "A161", "A163", "A164", "A165", "A166", "A168", "A865", "A130", "A131", "A133", "A134", "A135", "A136", "A138", "A435"
OHIP	DXCODE	"436", "432", "435"
Primary Care Visits		
IPDB	MAINSPECIALTY	"GP/FP"
OHIP	FEECODE	"00"
Cardiology Visits		
IPDB	MAINSPECIALTY	"CARDIOLOGY"
OHIP	FEECODE	"60", "09"
Teaching Hospital		
CIHI-DAD/SDS	INST	"1097", "1100", "1339", "1406", "1423", "1428", "1431", "1444", "1452", "1455", "1459", "1464", "1497", "1500", "1502", "1657", "1676", "1972", "1982", "1983", "1994", "2003", "3174", "3562", "3618", "3702", "3850", "3853", "3878", "3910", "3936", "4046", "4048", "4050", "4059", "4064", "4067", "4164", "4359", "4601", "4602"

Abbreviations: CIHI-DAD/SDS: Canadian Institutes for Health Information's Discharge Abstract Database/Same-Day Surgery, CCI: Canadian Classification of Interventions, CORR: Canadian Organ Replacement Registry, HYPER: Ontario Hypertension Dataset,

IPDB: ICES Physician Database, NACRS: National Ambulatory Care Reporting System, OHIP: Ontario Health Insurance Plan, OLIS: Ontario Laboratory Information System, RPDB: Registered Persons Database

Supplementary Table S4: Drugs from the Ontario Drug Benefit (ODB) database used to identify outcomes

Drug Class	Drug Name
Angiotensin-Converting Enzyme Inhibitor	Captopril, Enalapril,, Lisinopril, Hydrochlorothiazide & Lisinopril, Benazepril, Fosinopril, Cilazapril, Quinapril, Ramipril, Perindopril, Cilazapril & Hydrochlorothiazide, Trandolapril, Hydrochlorothiazide & Quinapril, Indapamide & Perindopril, Hydrochlorothiazide & Ramipril
Angiotensin II Receptor Blocker	Losartan, Valsartan, Irbesartan, Candesartan, Eprosartan, Telmisartan, Hydrochlorothiazide & Losartan, Hydrochlorothiazide & Irbesartan, Hydrochlorothiazide & Valsartan, Candesartan & Hydrochlorothiazide, Hydrochlorothiazide & Telmisartan, Eprosartan & Hydrochlorothiazide, Olmesartan, Hydrochlorothiazide & Olmesartan, Amlodipine & Telmisartan
Statins	Lovastatin, Simvastatin, Pravastatin, Fluvastatin, Atorvastatin, Cerivastatin, Rosuvastatin
Beta-Blocker	Propranolol, Timolol, Metoprolol, Oxprenolol, Pindolol, Nadolol, Hydrochlorothiazide & Propranolol, Sotalol, Atenolol, Hydrochlorothiazide & Pindolol, Labetalol, Atenolol & Chlorthalidone, Acebutolol, Carvedilol, Bisoprolol
Warfarin	Warfarin
Direct Oral Anticoagulant	Apixaban, Rivaroxaban, Dabigatran
P2Y12 inhibitors	Clopidogrel, Ticagrelor
Loop Diuretic	Bumetanide, Ethacrynic Acid, Furosemide
Thiazide Diuretic	Chlorthalidone, Hydrochlorothiazide, Hydrochlorothiazide & Timolol, Indapamide, Metolazone, Atenolol & Chlorthalidone, Indapamide & Perindopril, Hydrochlorothiazide & Methyldopa, Hydrochlorothiazide & Spironolactone, Hydrochlorothiazide & Triamterene, Hydrochlorothiazide & Propranolol HCL, Amiloride HCL & Hydrochlorothiazide, Hydrochlorothiazide & Pindolol, Hydrochlorothiazide & Lisinopril, Hydrochlorothiazide & Losartan, Hydrochlorothiazide & Quinapril, Hydrochlorothiazide & Irbesartan, Hydrochlorothiazide & Valsartan, Eprosartan & Hydrochlorothiazide, Hydrochlorothiazide & Ramipril, Cilazapril & Hydrochlorothiazide, Hydrochlorothiazide & Telmisartan, Hydrochlorothiazide & Olmesartan, Candesartan & Hydrochlorothiazide,
Mineralocorticoid Receptor Antagonist	Spironolactone, Hydrochlorothiazide & Spironolactone, Eplerenone
Dihydropyridine Calcium Channel Blocker	Nifedipine, Felodipine, Amlodipine, Amlodipine & Atorvastatin, Amlodipine & Telmisartan, Nicardipine
Biguanide	Metformin, Metformin & Sitagliptin, Metformin & Saxagliptin, Dapagliflozin & Metformin
Nonsteroidal-Anti-Inflammatory Drug	Phenylbutazone, Indomethacin, Mefenamic Acid, Ibuprofen, Naproxen, Fenoprofen, Ketoprofen, Tolmetin, Sulindac, Naproxen, Piroxicam, Diclofenac, Tiaprofenic Acid, Flurbiprofen, Floctafenine, Diclofenac & Misoprostol, Oxaprozin, Nabumetone, Etodolac, Ketorolac, Mefenamic Acid, Celecoxib, Valdecoxib, Meloxicam

Supplementary Table S5: Variables included in the propensity score model

Variables included in the propensity score model	
Demographics	Age, sex, year of cohort entry, income quintile, rural/urban location
Comorbidities	Charlson comorbidity score, acute dialysis, arrhythmia, atrial fibrillation, major cancers, cerebrovascular disease, chronic liver disease, chronic obstructive pulmonary disease, congestive heart failure, coronary artery bypass graft surgery, dementia, diabetes, gastrointestinal bleed, hypertension, pacemaker, percutaneous coronary intervention, peripheral vascular disease
Baseline Kidney Function	Proteinuria
Medications	Number of unique drugs prescribed (DINs), number of unique drugs prescribed (drug names), prescription for ACE/ARB, statins, and beta-blockers, DOACs, loop diuretics, thiazides, mineralocorticoid receptor antagonists, P2Y12 inhibitors, warfarin, metformin, NSAIDs, dihydropyridine calcium blockers
Health care use	Visits to general practitioner, visits to cardiologist, visits to nephrologist, number of hospitalizations, number of emergency department visits
Index hospitalizations characteristics	Teaching hospital, ICU/mechanical ventilation, CCU admit, cardiology involvement, sepsis, percutaneous coronary intervention, coronary artery bypass graft surgery, abdominal aortic aneurysm/aortic bypass repair, myocardial infarction, pacemaker, major hemorrhage, cerebrovascular disease, non-cardiac surgery, hospital length of day

Abbreviations: ACE- Angiotensin-Converting Enzyme, ARB- Angiotensin II Receptor Blocker, CCU-Cardiac Care Unit, DOAC- Direct Oral Anticoagulant, DIN- drug identification number; ICU- Intensive Care Unit, NSAID-Nonsteroidal-Anti-Inflammatory Drug

Supplementary Table S6: Unmatched characteristics of patients aged ≥ 66 years old who survived an inpatient hospitalization with a history of myocardial infarction prior to or during the index hospitalization

Baseline Characteristics	AKI Patients N=28,871	Hospitalized Controls N= 48,361	Standardized Difference
**Demographics, %**			
Age (years), mean (SD)	79.8 (8.0)	78.3 (8.1)	0.19
Female	11,587 (40.1)	19,594 (40.5)	0.01
Income Quintile			
1 (lowest income)	7,144 (24.7)	10,674 (22.1)	0.06
2	6,483 (22.5)	10,481 (21.7)	0.02
3 (mid income)	5,672 (19.6)	9,611 (19.9)	0.01
4	5,000 (17.3)	8,887 (18.4)	0.03
5 (highest income)	4,572 (15.8)	8,708 (18.0)	0.06
Rural Residence	4,196 (14.5)	7,652 (15.8)	0.04
**Comorbidities prior 5 years, %**			
Charlson comorbidity index, mean (SD)	1.8 (2.1)	0.9 (1.6)	0.47
Acute dialysis	416 (1.4)	214 (0.4)	0.10
Arrhythmia	8,724 (30.2)	9,383 (19.4)	0.25
Atrial fibrillation	8,978 (31.1)	10,279 (21.3)	0.23
Major cancer	5,742 (19.9)	9,091 (18.8)	0.03
Cerebrovascular disease	4,831 (16.7)	6,413 (13.3)	0.10
Chronic liver disease	1,769 (6.1)	2,091 (4.3)	0.08
Chronic obstructive pulmonary disease	4,274 (14.8)	4,192 (8.7)	0.19
Congestive heart failure	12,101 (41.9)	10,962 (22.7)	0.42
Coronary artery bypass graft surgery	1,186 (4.1)	1,657 (3.4)	0.04
Dementia	7,183 (24.9)	8,759 (18.1)	0.17
Diabetes	16,814 (58.2)	20,537 (42.5)	0.32
Gastrointestinal bleed	3,232 (11.2)	3,851 (8.0)	0.11
Hypertension	27,124 (93.9)	42,689 (88.3)	0.20
Pacemaker	3,194 (11.1)	3,470 (7.2)	0.14
Percutaneous coronary intervention	4,266 (14.8)	6,357 (13.1)	0.05
Peripheral vascular disease	1,795 (6.2)	1,697 (3.5)	0.13
**Baseline Kidney Function, %**			
Serum creatinine (mg/dL), mean (SD)	1.4 (0.8)	1.1 (0.5)	0.48
Pre-hospital eGFR			
≥60	10,447 (36.2)	29,411 (60.8)	0.51
45 to <60	6,790 (23.5)	10,411 (21.5)	0.05
30 to <45	6,778 (23.5)	6,120 (12.7)	0.28
<30	4,856 (16.8)	2,419 (4.4)	0.39

Proteinuria			
Normal	7,338 (25.4)	16,013 (33.1)	0.17
Moderate	4,296 (14.9)	4,720 (9.8)	0.16
Heavy	4,127 (14.3)	2,647 (5.5)	0.30
Missing	13,110 (45.4)	24,981 (51.7)	0.13
**Medication Use in prior 4 months, %**			
Unique drugs prescribed, mean (SD)	12.6 (5.8)	9.9 (5.3)	0.48
ACEi/ARB, Statins, and Beta-blocker	11,986 (41.5)	17,217 (35.6)	0.12
ACEi/ARB	20,727 (71.8)	32,307 (66.8)	0.11
Statins	21,514 (74.5)	33,930 (70.2)	0.10
Beta blockers	18,558 (64.3)	26,617 (55.0)	0.19
Loop diuretics	13,185 (45.7)	11,408 (23.6)	0.48
Thiazides	6,743 (23.4)	10,104 (20.9)	0.06
Mineralocorticoid receptor antagonists	2,907 (10.1)	2,385 (4.9)	0.20
Biguanides	7,451 (25.8)	10,231 (21.2)	0.11
P2Y12 inhibitors	7,125 (24.7)	9,503 (19.7)	0.12
Warfarin	4,582 (15.9)	5,031 (10.4)	0.16
DOACs	2,953 (10.2)	3,990 (8.3)	0.07
DHP Calcium Blockers	10,116 (35.0)	13,005 (26.9)	0.18
NSAIDs	2,546 (8.8)	5,348 (11.1)	0.07
**Health Care Utilization in the prior year, mean (SD)**			
Hospitalizations	1.3 (1.7)	0.8 (1.3)	0.31
Emergency department visits	2.2 (3.0)	1.5 (2.3)	0.26
Nephrology visits	0.5 (1.3)	0.2 (0.7)	0.34
Primary care visits	15.7 (14.9)	12.1 (11.6)	0.28
Cardiology visits	4.3 (6.7)	2.8 (4.5)	0.26
**Index Hospitalization Characteristics, %**			
Teaching Hospital	9,599 (33.2)	17,208 (35.6)	0.05
ICU/mechanical ventilation	9,552 (33.1)	14,208 (29.4)	0.08
CCU admit	1,719 (6.0)	2,569 (5.3)	0.03
Cardiology involvement	20,721 (71.8)	32,825 (67.9)	0.08
Myocardial infarction	9,779 (33.9)	17,093 (35.3)	0.03
Percutaneous coronary intervention	2,906 (10.1)	9,489 (19.6)	0.27
Coronary artery bypass graft surgery	1,770 (6.1)	2,817 (5.8)	0.01
Pacemaker	1,807 (6.3)	2,202 (4.6)	0.08
Abdominal aortic aneurysm/ aortic bypass repair	109 (0.4)	187 (0.4)	0.00
Major hemorrhage	1,604 (5.6)	1,767 (3.7)	0.09
Stroke/ TIA	1,763 (6.1)	3,511 (7.3)	0.05
Non-cardiac surgery	12,993 (45.0)	26,544 (54.9)	0.20
Sepsis	1,567 (5.4)	635 (1.3)	0.23

Length of stay (days), mean (SD)	14.3 (24.8)	7.9 (15.1)	0.31
Discharge potassium (mmol/L), mean (SD)	4.1 (0.5)	4.0 (0.4)	0.18
**AKI/Kidney Details**			
AKI severity			
Stage 1	23,678 (82.0)	0 (0.0)	3.02
Stage 2	3,177 (11.0)	0 (0.0)	0.50
Stage 3	2,016 (7.0)	0 (0.0)	0.39
Discharge creatinine, (mg/dL), mean (SD)	1.6 (1.1)	1.0 (0.4)	0.76
Discharge eGFR			
≥60	7,847 (27.2)	32,757 (67.7)	0.89
45 to <60	6,136 (21.3)	9,242 (19.1)	0.05
30 to <45	7,747 (26.8)	4,797 (9.9)	0.45
<30	7, 141 (24.7)	1, 565 (3.2)	0.65
Dialysis Dependence at discharge	376 (1.3)	0 (0.0)	0.16

Abbreviations: SD- Standard Deviation, eGFR- Estimated Glomerular Filtration Rate, ACE-Angiotensin- Converting Enzyme, ARB- Angiotensin II Receptor Blocker, DOAC- Direct Oral Anticoagulant, NSAID-Nonsteroidal-Anti-Inflammatory Drug, AKI- Acute Kidney Injury, ICU-Intensive Care Unit, CCU- Cardiac Care Unit, TIA- Transient Ischemic Attack

Supplementary Table S7: Association of different severities of AKI with receipt of other drug classes. The reference group for each stage of AKI is propensity-matched patients without AKI.

Outcome and Exposure	Events (n, %)	Sub-distribution Hazard Ratio (95% CI)	P-Value for Interaction
**Warfarin**			
Stage 1 AKI	3,167 (17.8)	1.16 (1.10-1.22)	p=0.7076
Control	2,768 (15.5)		
Stage 2 AKI	377 (15.8)	1.17 (1.01-1.35)	
Control	327 (13.7)		
Stage 3 AKI	200 (16.3)	1.26 (1.04-1.54)	
Control	161 (13.1)		
**DOAC**			
Stage 1 AKI	2,891 (16.2)	0.97 (0.92-1.02)	p=0.0025
Control	2, 894 (16.7)		
Stage 2 AKI	352 (4.7)	0.89 (0.77-1.02)	
Control	394 (16.5)		
Stage 3 AKI	120 (9.8)	0.65 (0.51-0.82)	
Control	180 (14.7)		
**P2Y12 inhibitors**			
Stage 1 AKI	6,483 (36.4)	0.99 (0.96-1.02)	p < 0.0001
Control	6, 535 (36.6)		
Stage 2 AKI	718 (30.0)	0.81 (0.74-0.89)	
Control	860 (36.0)		
Stage 3 AKI	379 (30.9)	0.71 (0.63-0.80)	
Control	496 (40.4)		
**Loop Diuretic**			
Stage 1 AKI	9,639 (54.1)	1.23 (1.20-1.26)	p < 0.0001
Control	8, 553 (46.8)		
Stage 2 AKI	1,145 (47.9)	1.11 (1.03-1.20)	
Control	1, 063 (44.5)		
Stage 3 AKI	521 (42.5)	0.93 (0.83-1.04)	
Control	548 (44.7)		
**Thiazide Diuretic**			
Stage 1 AKI	2,896 (16.2)	0.97 (0.92-1.02)	p=0.0023
Control	2, 983 (16.7)		
Stage 2 AKI	324 (13.6)	0.82 (0.71-0.94)	
Control	386 (16.1)		
Stage 3 AKI	138 (11.3)	0.70 (0.56-0.86)	
Control	192 (15.7)		
**Mineralocorticoid Receptor Antagonist**			
Stage 1 AKI	2,461 (13.8)	1.24 (1.17-1.32)	p=0.0553
Control	2, 006 (11.3)		
Stage 2 AKI	296 (12.4)	1.16 (0.98-1.36)	
Control	257 (10.8)		
Stage 3 AKI	117 (9.5)	0.92 (0.73-1.18)	
Control	125 (10.2)		
**DHP Calcium Channel Blocker**			

Stage 1 AKI	5,056 (28.4)	0.95 (0.91-0.98)	p=0.0429
Control	5,295 (29.7)		
Stage 2 AKI	594 (24.8)	0.82 (0.74-0.91)	
Control	702 (29.4)		
Stage 3 AKI	360 (29.3)	0.91 (0.80-1.05)	
Control	385 (31.4)		
**Biguanide**			
Stage 1 AKI	3,939 (22.1)	0.91 (0.88-0.94)	p=0.0002
Control	4, 297 (24.1)		
Stage 2 AKI	495 (20.7)	0.84 (0.76-0.92)	
Control	580 (24.3)		
Stage 3 AKI	232 (18.9)	0.68 (0.59-0.78)	
Control	325 (26.5)		
**NSAID**			
Stage 1 AKI	1,263 (7.1)	0.90 (0.84-0.97)	p=0.6015
Control	1, 397 (7.8)		
Stage 2 AKI	157 (6.6)	0.86 (0.70-1.06)	
Control	182 (7.6)		
Stage 3 AKI	65 (5.3)	0.77 (0.56-1.06)	
Control	84 (6.9)		

Abbreviations: DHP Dihydropyridine, DOAC- Direct Oral Anticoagulant, NSAID-Nonsteroidal-Anti-Inflammatory Drugs

Supplementary Table S8: Drug persistence and discontinuation at one-year.

	No. Patients	Mean days (SD)	Median days (IQR)	Discontinuation n (%)
**ACE/ARB**				
AKI	13,292	211.16 (127.62)	247 (88-337)	6013 (45.2)
Control	14,488	227.44 (123.84)	279 (101-340)	5822 (40.2)
**Beta-blocker**				
AKI	15,016	211.41 (129.70)	248 (80-339)	6611 (44.0)
Control	14,965	221.57 (127.38)	272 (91-340)	6189 (41.4)
**Statin**				
AKI	16,234	224.04 (125.70)	276 (92-339)	6697 (41.3)
Control	16,455	233.32 (122.96)	285 (105-341)	6311 (38.4)

To calculate duration of continuous use for each drug, we looked forward from the first prescription date during follow-up to 1.5 times the days supplied for a repeat study prescription. We continued looking forward from each repeat prescription until there were no more repeat prescriptions or until the end of the study period.
